# HLA Class I and II profiles in São Miguel Island (Azores): genetic diversity and linkage disequilibrium

**DOI:** 10.1186/1756-0500-3-134

**Published:** 2010-05-12

**Authors:** Paula R Pacheco, Claudia C Branco, Cidália T Gomes, Rita Cabral, Luisa Mota-Vieira

**Affiliations:** 1 Molecular Genetics and Pathology Unit, Hospital of Divino Espírito Santo of Ponta Delgada, EPE, São Miguel Island, Azores, Portugal; 2 Instituto Gulbenkian de Ciência, Oeiras, Portugal

## Abstract

**Background:**

Human leukocyte antigen (HLA) genes are characterized by high levels of polymorphism and linkage disequilibrium (LD), important characteristics to study the genetic background of human populations and their genetic structure. Here, we analyse the allele distribution and LD extent of HLA class I and II in São Miguel Island population (Azores archipelago, Portugal).

**Findings:**

The sample set was composed of 106 healthy blood donors living in São Miguel Island obtained from the anonymized Azorean DNA bank. HLA class I (-A, -B and -Cw) and class II (-DRB1, -DQB1, -DPA1 and -DPB1) genotyping was performed by PCR-SSP Olerup SSP™ (GenoVision Inc.), according to the manufacturer's instructions.

Genetic diversity values, based on the 7 *loci*, ranged from 0.821 both for HLA-DPA1 and -DQB1 to 0.934 for HLA-B, with a mean value of 0.846. Analysis of 5 HLA-A-Cw-B-DRB1-DQB1 haplotypes revealed that A*01-Cw*07-B*08-DRB1*03-DQB1*02 is the most frequent in São Miguel (7.9%) followed by A*24-B*08-Cw*07-DRB1*03-DQB1*02 (3.8%). In addition, even though the reports of high LD for HLA markers in worldwide populations, São Miguel islanders do not have extensive LD (average D' = 0.285).

**Conclusions:**

In summary, the results demonstrate high variability of HLA in São Miguel Island population as well as absence of genetic structure and extensive LD. The data here presented suggest that in São Miguel islanders autoimmune diseases studies will necessarily encompass a more focused analysis of HLA extended haplotypes as well as the evaluation of other non-HLA candidate genes.

## Background

The Azores is a Portuguese archipelago composed of nine islands distributed by three geographical groups: the Eastern (São Miguel and Santa Maria), the Central (Terceira, Pico, Faial, São Jorge and Graciosa) and the Western (Flores and Corvo). The Portuguese explorers, who discovered the archipelago in 1427, only started the settlement in 1439 through a long and difficult process. Historical data report a contribution from people with genetic backgrounds other than Portuguese, including Flemish, Spanish, French, Italian, German, Scottish, Jewish, and also from Moorish prisoners and black slaves from Guinea, Cape Verde and São Tomé [1]. São Miguel is the largest island of the Azores and is composed of 131,609 inhabitants (2001 Census, Portugal National Institute of Statistics). Several studies have been performed to characterize the genetic pool of the Azoreans [2-10]. These studies report a high genetic variability and heterogeneity of the Azorean population, explained by the settling history of the islands, where a major contribution of mainland Portugal individuals is evident. Moreover, the data revealed absence of population structure, even though the archipelago's geographical discontinuity and demographic disproportionality. Currently, this knowledge is being fundamental for the design and development of pharmacogenetic research and genetic studies in common diseases, such as cardiovascular and autoimmune diseases.

The human leukocyte antigen (HLA) genes, a central component of the major histocompatibility complex (MHC) on 6p21.3, encode polymorphic class I, II and III molecules that play a major role in the immune response [11]. In addition, HLA *loci *are characterized by high levels of polymorphism and linkage disequilibrium (LD), important characteristics to study the genetic background of human populations, as well as their present-day genetic structure. Here, we analyse the allele frequency and LD extent of HLA class I and II, in order to identify its diversity and haplotype distribution and to gain further insight in the potential use of this genomic region for the study of autoimmune diseases in the São Miguel Island population.

## Materials and methods

### Population samples, genotyping and statistical analysis

The sample set was composed of 106 healthy blood donors living in São Miguel Island obtained from the anonymized Azorean DNA bank located at the Hospital of Divino Espírito Santo of Ponta Delgada, EPE, the main hospital in Azores [12]. HLA class I (-A, -Cw and -B) and class II (-DRB1, -DQB1, -DPA1 and -DPB1) genotyping was performed by PCR-SSP Olerup SSP™ (GenoVision Inc.), according to the manufacturer's instructions. After electrophoresis on a 4% agarose gel stained with SYBR^® ^Green, PCR products were visualized, followed by HLA allele identification using the Helmberg-SCORE™ software version 3.320T (Olerup SSP AB, Saltsjöbaden, Sweden).

Average gene diversity and estimation of the HLA haplotypes was carried out using Arlequin v3.0 [13]. Evaluation of standardized multiallelic disequilibrium coefficient, D', was performed by the Haploxt application from the GOLD software. Average D' values were calculated by a simple mathematical mean of all values obtained for each marker pair. Nei's F_ST _genetic distance matrix was computed between pairs of populations by DISPAN [14] and used to construct a Neighbor-Joining (NJ) tree by PHYLIP 3.63 [15]. We employed TreeView 1.6.6 [16] to display tree phylogenies obtained from NJ. In order to obtain the best results concerning population comparisons a compromise between the number of populations and HLA *loci *was performed. Consequently, HLA-DPA1 and -DPB1 were excluded from analysis. F_ST _values were based on allele frequencies obtained in an online database (HLA-Allele Project; http://www.allelefrequencies.net/), in 19 populations for 5 HLA *loci*: São Miguel, Terceira, Italy, France, Germany, Belgium, Turkey, Morocco, Japan, Mongolia Oold, Mongolia Tsaatan, Mongolia Khalkha, Basque, Ibiza, Majorca, Majorca Jewish, Chuetas, Minorca and Jordania. Along with F_ST _values, 5 *loci *haplotypes were searched in the same database to further investigate the possible origins of the early settlers.

## Results

The analysis of the HLA alleles in the São Miguel Island population (Table 1) revealed for the HLA-A *locus *a total of 16 different alleles, 13 HLA-Cw and 24 HLA-B alleles. Regarding HLA class II *loci*, we found 22 HLA-DPB1, 13 HLA-DRB1, 5 HLA-DQB1 and 6 HLA-DPA1 different alleles. HLA-B and HLA-DPB1 are the two *loci *with the highest numbers of alleles, suggesting higher diversity for these markers. The highest frequency observed, 0.462, was in HLA-DPA1 gene, which shows a low number of alleles. In contrast, the lowest frequency identified (0.5%) was present in HLA-A, -B and -DPB1 (Table 1). Genetic diversity values ranged from 0.821 both for HLA-DPA1 and -DQB1 to 0.934 for HLA-B, with a mean value of 0.846 (Table 2). Overall, HLA allele frequencies in São Miguel, mainland Portugal and other European populations demonstrated absence of statistically significant differences (G_ST _= 0.03; data not shown). According to Wright [17] values of G_ST _smaller than 0.05 indicate little genetic differentiation.

**Table 1:** HLA class I and II allele frequencies in São Miguel population (the highest values are in bold).

**HLA class I (2n = 212)**				**HLA class II (2n = 212)**			
	
**Alleles**	**Rel. Freq.**	**Alleles**	**Rel. Freq.**	**Alleles**	**Rel. Freq.**	**Alleles**	**Rel. Freq.**
			
**HLA-A**		**HLA-B**		**HLA-DPB1**		**HLA-DRB1**	
			
A*01	0.151	B*07	0.066	DPB1*0101	0.057	DRB1*01	0.085
A*02	**0.250**	B*08	0.137	DPB1*0201	0.212	DRB1*03	0.165
A*03	0.094	B*13	0.005	DPB1*0202	0.014	DRB1*04	0.123
A*11	0.042	B*14	0.071	DPB1*0301	0.080	DRB1*07	**0.170**
A*23	0.019	B*15	0.052	DPB1*0401	**0.316**	DRB1*08	0.028
A*24	0.137	B*18	0.052	DPB1*0402	0.094	DRB1*09	0.019
A*25	0.005	B*27	0.042	DPB1*0501	0.014	DRB1*10	0.019
A*26	0.009	B*35	0.061	DPB1*0601	0.005	DRB1*11	0.118
A*29	0.066	B*37	0.014	DPB1*0901	0.005	DRB1*12	0.009
A*30	0.033	B*38	0.014	DPB1*1001	0.028	DRB1*13	0.146
A*31	0.024	B*39	0.009	DPB1*1101	0.024	DRB1*14	0.019
A*32	0.061	B*40	0.028	DPB1*1301	0.052	DRB1*15	0.075
A*33	0.028	B*41	0.024	DPB1*1401	0.014	DRB1*16	0.024
						
A*66	0.005	B*44	**0.156**	DPB1*1501	0.005	**HLA-DQB1**	
						
A*68	0.071	B*45	0.009	DPB1*1601	0.005	DQB1*02	0.302
A*80	0.005	B*47	0.005	DPB1*1701	0.038	DQB1*03	**0.321**
						
**HLA-Cw**		B*49	0.052	DPB1*1901	0.014	DQB1*04	0.028
						
Cw*01	0.024	B*50	0.033	DPB1*2501	0.005	DQB1*05	0.151
Cw*02	0.066	B*51	0.066	DPB1*3901	0.005	DQB1*06	0.198
Cw*03	0.075	B*53	0.024	DPB1*5101	0.005		
Cw*04	0.104	B*55	0.019	DPB1*6601	0.005		
Cw*05	0.071	B*57	0.042	DPB1*7801	0.005		
						
Cw*06	0.090	B*58	0.014	**HLA-DPA1**			
						
Cw*07	**0.311**	B*78	0.005	DPA1*01	**0.462**		
Cw*08	0.052			DPA1*0103	0.255		
Cw*12	0.047			DPA1*0105	0.005		
Cw*14	0.019			DPA1*0201	0.226		
Cw*15	0.047			DPA1*0202	0.042		
Cw*16	0.071			DPA1*0301	0.009		
Cw*17	0.024						

**Table 2:** Gene diversity (GD) and linkage disequilibrium (D') values for 7 HLA *loci *in São Miguel Island population.

Gene diversity		Linkage disequilibrium	
	
HLA *loci*	GD	HLA *loci *pair	D'
	
A	0.877	A-Cw	0.301
		A-B	0.317
		A-DRB1	0.231
		A-DQB1	0.207
		A-DPA1	0.172
		A-DPB1	0.175
B	**0.934**	B-Cw	0.571
		B-DRB1	0.341
		B-DQB1	0.258
		B-DPA1	0.221
		B-DPB1	0.205

Cw	0.839	Cw-DRB1	0.356
		Cw-DQB1	0.253
		Cw-DPA1	0.275
		Cw-DPB1	0.164

DPA1	**0.821**	DPA1-DPB1	0.398
		DPA1-DQB1	**0.163**
		DPA1-DRB1	0.270

DPB1	0.906	DPB1-DQB1	0.191
		DPB1-DRB1	0.213

DQB1	**0.821**	DQB1-DRB1	**0.712**

DRB1	0.877	**--**	**--**

**Average GD**	**0.846**	**Average D'**	**0.285**

In bold are the highest and the smallest values.

Considering the 7 HLA *loci*, haplotype determination demonstrates a total of 176 different haplotypes corresponding to an 83.0% discriminatory power. Analysis of 5 HLA-A-Cw-B -DRB1-DQB1 haplotypes was also performed (see Additional file 1 for details). The results indicate that A*01-Cw*07-B*08-DRB1*03-DQB1*02 is the most frequent in São Miguel (7.9%), followed by A*24-Cw*07-B*08-DRB1*03-DQB1*02 (3.8%). Both A*02-Cw*05-B*44-DRB1*04-DQB1*03 and A*29-Cw*16-B*44-DRB1*07-DQB1*02 are present at a frequency of 1.9%. A total of 157 haplotypes were matched against worldwide populations (HLA-Allele Project; http://www.allelefrequencies.net/). The results showed that the second most frequent haplotype, above described, appears only on Tunisia. Moreover, just 9 haplotypes (Haplotype number - HN - 1, 29, 37, 42, 84, 85, 101, 104 and 112; see Additional file 1 for details) were identified in this database.

Linkage disequilibrium was based on the calculation of standardized multiallelic disequilibrium coefficient, D'. The range values are 0.163 for HLA markers DPA1-DQB1 and 0.712 for DQB1-DRB1 (Table 2). This wide variation averages 0.285 for the 7 *loci*. Curiously, the genetically closest markers (DPA1-DPB1, 0.011 Mb; D' = 0.398) do not present the highest value of D' (DQB1-DRB1, 0.081 Mb; D' = 0.712). A poor correlation between distance (Mb) and D' is observed, although there is a decrease of LD values over physical distance increase, as expected.

**Figure 1:**
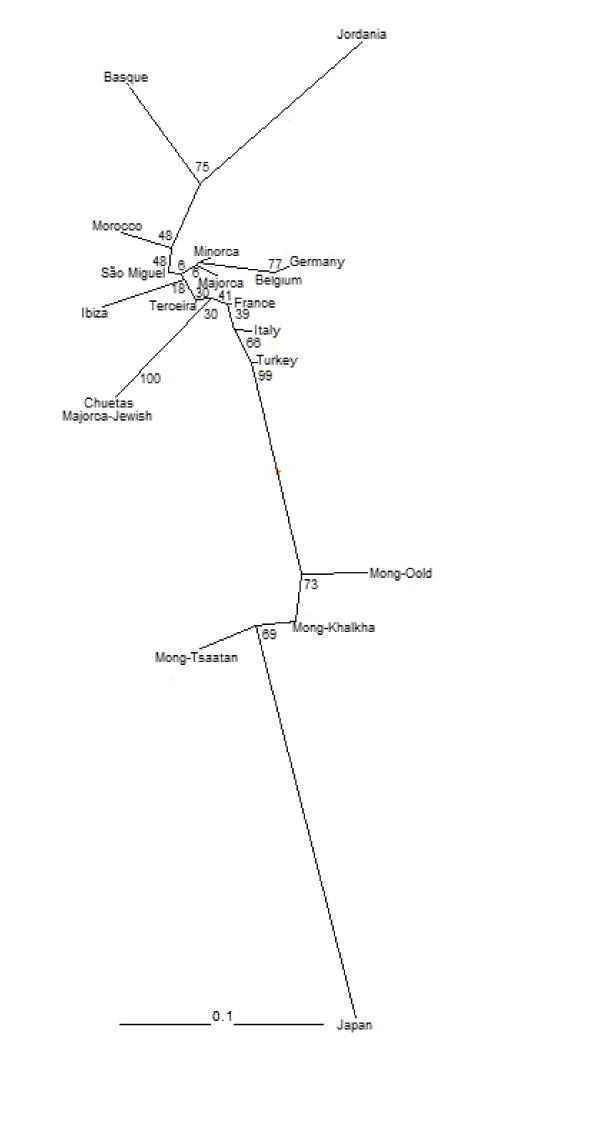
**Neighbor-Joining tree comparing 5 HLA *loci *in 19 populations.** São Miguel clusters with Europeans and Moroccans.

In order to obtain a graphical view of the genetic similarity between São Miguel (106 individuals, 5 HLA *loci*) and other populations, we computed Nei's genetic distances and depicted them in Figure 1. Interestingly, São Miguel is closer to Morocco population than to Terceira, another Azorean island. Nevertheless, in general, both populations cluster within the Europeans.

## Discussion

Extensive studies have been performed in several geographical areas to characterize the diversity of HLA genetic markers. These evaluations allow a better knowledge of the population structure considering non-neutral markers, as well as an understanding of the influence of evolutionary processes in the overall signature of a population. These genetic data are crucial for the comprehension of the molecular ethiology and epidemiology of common diseases. In general, the data here presented corroborate previous works [3,6-10], where Azoreans including São Miguel islanders show high values of genetic diversity when compared to mainland Portugal and other European populations. This may be a direct consequence of the Azorean settlement, with a major contribution of mainland Portuguese (~60%) and, to a lesser extent, Flemish, Spanish, French, Italians, Germans, Scottish, Jews, Moors and blacks from Guinea, Cabo Verde and São Tomé. Previous studies of HLA markers in mainland Portugal (3 *loci*, -A, -B and -DRB1, [18]) and in Azores (6 *loci*, -A, -Cw, -B, -DRB1, -DQA1 and -DQB1, [5]) demonstrate values of average diversity of 0.92 in both populations. The results obtained in the present study, based in 7 *loci*, showed a smaller value (0.84). This may be explained by the fact that Spinola et al. [5] used a high-resolution methodology to genotype HLA. Because alleles A*0101 and A*0102 are not considered the same allele (A*01), this methodology allows the identification of a higher number of different alleles. Nonetheless, the data show no significant differences between allele frequencies in São Miguel and Terceira islands. Considering HLA alleles distribution, the presence of -A*30 and -A*80, commonly found in sub-Saharan populations [19-21], in São Miguel validates historic records of slave settlers. In addition, the presence of alleles -B*35, -B*57 and -B*15 suggest a direct contribution of Moorish prisoners in Azores [22-24]. Nevertheless, the influence of early Portuguese settlers can not be ruled out since allele frequencies are similar. In general, these results are corroborated by the NJ tree (Figure 1), where São Miguel shows influence of both African and European populations.

Linkage disequilibrium is considered a good measure of population structure. According to Sanchez-Mazas [25] HLA-DPB1, located on the centromeric side of the HLA chromosomal region, does not show high values of LD with the other HLA *loci*. Interestingly, in the present study, the lowest values of D' observed are related with this marker. This result is explained by the high recombination region involving one or several hotspots, which separates HLA-DPB1 from the rest of the other HLA *loci*. Abecasis et al. [26] discuss that a value of D' = 0.33, which corresponds to a 10-fold increase in the required sample size, is commonly taken as the minimum usable amount of LD. Considering the 21 possible HLA *loci *combinations, 17 demonstrated values inferior to 0.33, and only 2 (Cw-B and DQB1-DRB1) showed values significantly higher (0.571 and 0.712, respectively). The HLA data reported by Meyer et al. [27] indicate a significant LD between all HLA *loci *in around 40 worldwide studied populations. The present research did not indicate large D' values and corroborates the results obtained by Service et al. [28] and Branco et al. [9,10], where the Azoreans have the lowest values of LD when compared with isolated and outbred populations.

HLA diversity in human populations is an important aspect of disease epidemiology, especially autoimmune disorders, such as type I diabetes, ankylosing spondylitis and celiac disease. According to Bakker et al. [29], the association of HLA alleles and/or haplotypes with disease susceptibility may be confounded by the presence of population stratification in neighboring HLA and non-HLA genomic regions. The high variability of HLA, and the absence of genetic structure and extensive LD, here demonstrated, suggest that autoimmune diseases studies in São Miguel islanders will necessarily encompass a more focused analysis of HLA extended haplotypes, as well as the evaluation of other non-HLA candidate genes.

## Competing interests

The authors declare that they have no competing interests.

## Authors' contributions

PRP and CCB, contributed equally, by performing the experiments, statistical analysis and drafting the manuscript. CTG and RC genotyped individuals from the patients sample and provide technical help, respectively. LMV provided scientific orientation and revised the manuscript. All authors read and approved the final manuscript.

## Supplementary Material

Additional file 1 **Supplemental data to Results**. Details each haplotype found in the São Miguel Island considering 5 HLA *loci *(A*-Cw*B*-DRB1*-DQB1) as well as their relative frequency. Click here for file

## References

[B1] Guill JH A history of the Azores islands 1993 California, Division of Golden Shield International Publications Cooperation

[B2] Santos C Lima M Montiel R Angles N Pires L Abade A Aluja MP Genetic structure and origin of peopling in the Azores islands (Portugal): The view from mtDNA Ann Hum Genet 2003 67 433 456 10.1046/j.1469-1809.2003.00031.x 12940917

[B3] Pacheco PR Branco CC Cabral R Costa S Araújo AL Peixoto BR Mendonça P Mota-Vieira L The Y-chromosomal heritage of the Azores Islands population Ann Hum Genet 2005 69 145 156 10.1046/j.1469-1809.2004.00147.x 15720296

[B4] Montiel R Bettencourt C Silva C Santos C Prata MJ Lima M Analysis of Y-chromosome variability and its comparison with mtDNA variability reveals different demographic histories between islands in the Azores Archipelago (Portugal) Ann Hum Genet 2005 69 135 144 10.1046/j.1469-1809.2004.00146.x 15720295

[B5] Spínola H Brehm A Bettencourt B Middleton D Bruges-Armas J HLA class I and II polymorphisms in Azores show different settlements in Oriental and Central islands Tissue Antigens 2005 66 217 230 10.1111/j.1399-0039.2005.00471.x 16101833

[B6] Branco CC Palla R Lino S Pacheco PR Cabral R de Fez L Peixoto BR Mota-Vieira L Assessment of the Azorean ancestry by *Alu *insertion polymorphisms Am J Hum Biol 2006 18 223 226 10.1002/ajhb.20492 16493635

[B7] Branco CC São-Bento M Gomes CT Cabral R Pacheco PR Mota-Vieira L Azores Islands: genetic origin, gene flow and diversity patterns Ann Hum Biol 2008 35 65 74 10.1080/03014460701793782 18274926

[B8] Branco CC Pacheco PR Cabral R Vicente AM Mota-Vieira L Genetic signature of the São Miguel Island population (Azores) assessed by 21 microsatellite *loci* Am J Hum Biol 2008 20 118 120 10.1002/ajhb.20692 17990326

[B9] Branco CC Cabrol E São Bento M Gomes CT Cabral R Vicente AM Pacheco PR Mota-Vieira L Evaluation of linkage disequilibrium on the Xq13.3 region: comparison between the Azores Islands and mainland Portugal Am J Hum Biol 2008 20 364 366 10.1002/ajhb.20734 18257075

[B10] Branco CC Pacheco PR Cabrol E Cabral R Vicente AM Mota Vieira L Linkage disequilibrium and diversity for three genomic regions in Azoreans and mainland Portuguese Genet Mol Biol 2009 in press 21637671 10.1590/S1415-47572009000200003 PMC3036928

[B11] van Oosterhout C A new theory of MHC evolution: beyond selection on the immune genes Proc Biol Sci 2009 276 657 665 10.1098/rspb.2008.1299 18986972 PMC2660941

[B12] Mota-Vieira L Pacheco PR Almeida ML Cabral R Carvalho J Branco CC de Fez L Peixoto BR Araújo AL Mendonça P Amorim A, Côrte-Real F, Morling N Human DNA bank in São Miguel island (Azores), a resource for genetic diversity studies Progress in Forensic Genetics, Proceedings of the 21st International Congress of Forensic Genetics, 13 - 17 September Ponta Delgada 2005 1288 388 390

[B13] Schneider S Roessli D Excoffier L Arlequin: A software for population genetics data analysis 2000 University of Geneva, Genetics and Biometry Laboratory. Geneva

[B14] Ota T DISPAN: Genetic distance and phylogenetic analysis Institute of Molecular Evolutionary Genetics 1993 The Pennsylvania State University, USA

[B15] Felsenstein J PHYLIP (Phylogeny Inference Package) version 35c 1993 Distributed by the author. Department of Genetics, University of Washington, Seattle, WA

[B16] Page RDM TREEVIEW: An application to display phylogenetic trees on personal computers Computer Applications in the Biosciences 1996 12 357 358 8902363 10.1093/bioinformatics/12.4.357

[B17] Wright S Evolution and the genetics of populations: Variability within and among natural populations 1984 Chicago: Chicago University Press

[B18] Spínola H Middleton D Brehm A HLA genes in Portugal inferred from sequence-based typing: in the crossroad between Europe and Africa Tissue Antigens 2005 66 26 36 10.1111/j.1399-0039.2005.00430.x 15982254

[B19] Arnaiz-Villena A Elaiwa N Silvera C Rostom A Moscoso J Gómez-Casado E Allende L Varela P Martínez-Laso J The origin of Palestinians and their genetic relatedness with other Mediterranean populations Hum Immunol 2001 62 889 900 10.1016/S0198-8859(01)00288-9 11543891

[B20] Arnaiz-Villena A Gomez-Casado E Martinez-Laso J Population genetic relationships between Mediterranean populations determined by HLA allele distribution and a historic perspective Tissue Antigens 2002 60 111 121 10.1034/j.1399-0039.2002.600201.x 12392505

[B21] Gómez-Casado E del Moral P Martínez-Laso J García-Gómez A Allende L Silvera-Redondo C Longas J González-Hevilla M Kandil M Zamora J Arnaiz-Villena A HLA genes in Arabic-speaking Moroccans: close relatedness to Berbers and Iberians Tissue Antigens 2000 55 239 249 10.1034/j.1399-0039.2000.550307.x 10777099

[B22] Muro M Marín L Torío A Moya-Quiles MR Minguela A Rosique-Roman J Sanchis MJ Garcia-Calatayud MC García-Alonso AM Alvarez-López MR HLA polymorphism in the Murcia population (Spain): in the cradle of the archaeologic Iberians Hum Immunol 2001 62 910 921 10.1016/S0198-8859(01)00290-7 11543893

[B23] Modiano D Luoni G Petrarca V Sodiomon Sirima B De Luca M Simporé J Coluzzi M Bodmer JG Modiano G HLA class I in three West African ethnic groups: genetic distances from sub-Saharan and Caucasoid populations Tissue Antigens 2001 57 128 137 10.1034/j.1399-0039.2001.057002128.x 11260507

[B24] Bera O Cesaire R Quelvennec E Quillivic F de Chavigny V Ribal C Semana G HLA class I and class II allele and haplotype diversity in Martinicans Tissue Antigens 2001 57 200 207 10.1034/j.1399-0039.2001.057003200.x 11285127

[B25] Sanchez-Mazas A An apportionment of human HLA diversity Tissue Antigens 2007 69 198 202 10.1111/j.1399-0039.2006.00802.x 17445200

[B26] Abecasis GR Noguchi E Heinzmann A Traherne JA Bhattacharyya S Leaves NI Anderson GG Zhang Y Lench NJ Carey A Cardon LR Moffatt MF Cookson WO Extent and distribution of linkage disequilibrium in three genomic regions Am J Hum Genet 2001 68 191 197 10.1086/316944 11083947 PMC1234912

[B27] Meyer D Single RM Mack SJ Erlich HA Thomson G Signatures of demographic history and natural selection in the human major histocompatibility complex *loci* Genetics 2006 173 2121 2142 10.1534/genetics.105.052837 16702436 PMC1569707

[B28] Service S DeYoung J Karayiorgou M Roos JL Pretorious H Bedoya G Ospina J Ruiz-Linares A Macedo A Palha JA Heutink P Aulchenko Y Oostra B van Duijn C Jarvelin MR Varilo T Peddle L Rahman P Piras G Monne M Murray S Galver L Peltonen L Sabatti C Collins A Freimer N Magnitude and distribution of linkage disequilibrium in population isolates and implications for genome-wide association studies Nat Genet 2006 38 556 560 10.1038/ng1770 16582909

[B29] de Bakker PI McVean G Sabeti PC Miretti MM Green T Marchini J Ke X Monsuur AJ Whittaker P Delgado M Morrison J Richardson A Walsh EC Gao X Galver L Hart J Hafler DA Pericak-Vance M Todd JA Daly MJ Trowsdale J Wijmenga C Vyse TJ Beck S Murray SS Carrington M Gregory S Deloukas P Rioux JD A high-resolution HLA and SNP haplotype map for disease association studies in the extended human MHC Nat Genet 2006 38 1166 1172 10.1038/ng1885 16998491 PMC2670196

