# Supplementary material for: HLA Class I and II profiles in São Miguel Island (Azores): genetic diversity and linkage disequilibrium
Source: BMC Res Notes. 2010 May 12;3:134. doi: 10.1186/1756-0500-3-134 (PMC2883542; doi:10.1186/1756-0500-3-134)
Supplement: Additional file 1 — Supplemental data to Results. Details each haplotype found in the São Miguel Island considering 5 HLA loci (A*-Cw*B*-DRB1*-DQB1) as well as their relative frequency. [file 1756-0500-3-134-S1.DOC]

**Supplemental data to Results**

**Table S1**. Haplotype analysis considering 5 HLA *loci* (A*-Cw*B*-DRB1*-DQB1). In bold are the haplotypes with frequency higher than 1%.

| **Haplotype Nº** | **Relative frequency (%)** | **HLA alleles** | | | | |
| --- | --- | --- | --- | --- | --- | --- |
|  |  | **A*** | **Cw*** | **B*** | **DRB1*** | **DQB1*** |
| **1** | **7.90** | **01** | **07** | **08** | **03** | **02** |
| 2 | 0.47 | 01 | 07 | 08 | 13 | 06 |
| 3 | 0.47 | 01 | 03 | 15 | 04 | 05 |
| 4 | 0.47 | 01 | 01 | 27 | 13 | 03 |
| 5 | 0.47 | 01 | 03 | 40 | 15 | 03 |
| 6 | 0.47 | 01 | 16 | 44 | 07 | 06 |
| 7 | 0.59 | 01 | 06 | 50 | 07 | 02 |
| 8 | 0.47 | 01 | 05 | 51 | 04 | 03 |
| 9 | 0.47 | 01 | 14 | 51 | 07 | 02 |
| 10 | 0.47 | 01 | 14 | 51 | 15 | 04 |
| 11 | 0.47 | 01 | 06 | 57 | 12 | 03 |
| **12** | **1.42** | **01** | **07** | **57** | **07** | **03** |
| 13 | 0.47 | 01 | 07 | 57 | 11 | 03 |
| 14 | 0.47 | 01 | 07 | 08 | 03 | 02 |
| 15 | 0.94 | 02 | 07 | 07 | 08 | 06 |
| 16 | 0.47 | 02 | 07 | 07 | 15 | 03 |
| 17 | 0.47 | 02 | 15 | 07 | 16 | 05 |
| 18 | 0.47 | 02 | 07 | 08 | 03 | 02 |
| 19 | 0.47 | 02 | 05 | 13 | 03 | 03 |
| 20 | 0.94 | 02 | 02 | 14 | 01 | 05 |
| **21** | **1.42** | **02** | **08** | **14** | **07** | **02** |
| 22 | 0.47 | 02 | 03 | 15 | 04 | 06 |
| 23 | 0.47 | 02 | 07 | 15 | 09 | 03 |
| 24 | 0.47 | 02 | 07 | 15 | 13 | 06 |
| 25 | 0.47 | 02 | 05 | 18 | 04 | 03 |
| 26 | 0.47 | 02 | 05 | 18 | 15 | 03 |
| 27 | 0.94 | 02 | 07 | 18 | 11 | 03 |
| **28** | **1.42** | **02** | **04** | **35** | **01** | **05** |
| 29 | 0.47 | 02 | 12 | 38 | 11 | 03 |
| 30 | 0.47 | 02 | 03 | 40 | 04 | 02 |
| 31 | 0.47 | 02 | 03 | 40 | 13 | 03 |
| 32 | 0.94 | 02 | 02 | 41 | 13 | 06 |
| 33 | 0.47 | 02 | 04 | 41 | 01 | 04 |
| 34 | 0.47 | 02 | 03 | 44 | 11 | 03 |
| 35 | 0.47 | 02 | 04 | 44 | 07 | 02 |
| 36 | 0.47 | 02 | 04 | 44 | 13 | 03 |
| **37** | **1.89** | **02** | **05** | **44** | **04** | **03** |
| 38 | 0.47 | 02 | 05 | 44 | 07 | 02 |
| 39 | 0.47 | 02 | 05 | 44 | 13 | 06 |
| 40 | 0.47 | 02 | 05 | 44 | 15 | 06 |
| 41 | 0.47 | 02 | 15 | 44 | 01 | 05 |
| 42 | 0.47 | 02 | 16 | 44 | 07 | 02 |
| 43 | 0.47 | 02 | 16 | 44 | 11 | 03 |
| 44 | 0.47 | 02 | 16 | 44 | 13 | 06 |

**Table S1**. *(cont.)*

| **Haplotype Nº** | **Relative frequency (%)** | **HLA alleles** | | | | |
| --- | --- | --- | --- | --- | --- | --- |
|  |  | **A*** | **Cw*** | **B*** | **DRB1*** | **DQB1*** |
| 45 | 0.94 | 02 | 06 | 49 | 07 | 03 |
| 46 | 0.47 | 02 | 06 | 50 | 03 | 05 |
| 47 | 0.47 | 02 | 01 | 51 | 13 | 06 |
| **48** | **1.42** | **02** | **04** | **51** | **11** | **03** |
| 49 | 0.47 | 02 | 16 | 51 | 04 | 03 |
| 50 | 0.47 | 02 | 16 | 51 | 07 | 02 |
| 51 | 0.47 | 02 | 04 | 53 | 04 | 03 |
| 52 | 0.47 | 02 | 03 | 55 | 08 | 04 |
| 53 | 0.47 | 02 | 03 | 58 | 03 | 02 |
| 54 | 0.47 | 03 | 07 | 07 | 08 | 03 |
| 55 | 0.47 | 03 | 07 | 07 | 11 | 03 |
| 56 | 0.47 | 03 | 07 | 07 | 16 | 05 |
| 57 | 0.47 | 03 | 15 | 07 | 04 | 03 |
| 58 | 0.94 | 03 | 15 | 14 | 01 | 05 |
| 59 | 0.47 | 03 | 03 | 15 | 15 | 04 |
| 60 | 0.47 | 03 | 02 | 18 | 13 | 06 |
| 61 | 0.47 | 03 | 03 | 18 | 14 | 05 |
| 62 | 0.47 | 03 | 05 | 18 | 13 | 06 |
| 63 | 0.47 | 03 | 07 | 18 | 11 | 03 |
| 64 | 0.47 | 03 | 02 | 27 | 11 | 03 |
| 65 | 0.47 | 03 | 16 | 35 | 11 | 03 |
| 66 | 0.47 | 03 | 16 | 44 | 04 | 03 |
| 67 | 0.47 | 03 | 16 | 45 | 10 | 05 |
| 68 | 0.94 | 03 | 07 | 49 | 13 | 06 |
| 69 | 0.47 | 03 | 12 | 55 | 11 | 03 |
| 70 | 0.94 | 03 | 06 | 57 | 07 | 02 |
| 71 | 0.47 | 11 | 06 | 27 | 11 | 02 |
| 72 | 0.47 | 11 | 04 | 35 | 04 | 04 |
| 73 | 0.47 | 11 | 02 | 40 | 09 | 03 |
| 74 | 0.47 | 11 | 03 | 40 | 04 | 06 |
| 75 | 0.47 | 11 | 08 | 40 | 01 | 05 |
| 76 | 0.47 | 11 | 02 | 44 | 16 | 05 |
| 77 | 0.47 | 11 | 04 | 44 | 12 | 03 |
| 78 | 0.47 | 11 | 15 | 44 | 04 | 03 |
| 79 | 0.47 | 11 | 03 | 55 | 14 | 05 |
| 80 | 0.47 | 23 | 17 | 14 | 01 | 05 |
| 81 | 0.47 | 23 | 04 | 44 | 04 | 02 |
| 82 | 0.47 | 23 | 07 | 49 | 08 | 03 |
| 83 | 0.47 | 23 | 07 | 49 | 13 | 06 |
| 84 | 0.94 | 24 | 07 | 07 | 15 | 06 |
| **85** | **3.82** | **24** | **07** | **08** | **03** | **02** |
| 86 | 0.55 | 24 | 08 | 14 | 07 | 02 |
| **87** | **1.42** | **24** | **08** | **14** | **15** | **06** |
| 88 | 0.94 | 24 | 03 | 15 | 11 | 06 |
| 89 | 0.47 | 24 | 03 | 15 | 13 | 03 |
| 90 | 0.47 | 24 | 01 | 27 | 01 | 05 |
| 91 | 0.47 | 24 | 04 | 27 | 03 | 02 |
| 92 | 0.47 | 24 | 12 | 35 | 03 | 02 |
| 93 | 0.47 | 24 | 14 | 35 | 07 | 03 |

**Table S1**. *(cont.)*

| **Haplotype Nº** | **Relative frequency (%)** | **HLA alleles** | | | | |
| --- | --- | --- | --- | --- | --- | --- |
|  |  | **A*** | **Cw*** | **B*** | **DRB1*** | **DQB1*** |
| 94 | 0.47 | 24 | 04 | 38 | 13 | 05 |
| 95 | 0.47 | 24 | 02 | 44 | 03 | 02 |
| 96 | 0.47 | 24 | 05 | 44 | 13 | 06 |
| 97 | 0.82 | 24 | 06 | 50 | 07 | 02 |
| 98 | 0.47 | 24 | 05 | 51 | 14 | 05 |
| 99 | 0.47 | 24 | 15 | 51 | 14 | 03 |
| 100 | 0.47 | 24 | 07 | 57 | 13 | 06 |
| 101 | 0.47 | 25 | 12 | 18 | 15 | 06 |
| 102 | 0.47 | 26 | 08 | 47 | 07 | 02 |
| 103 | 0.47 | 26 | 14 | 53 | 15 | 06 |
| 104 | 0.55 | 29 | 07 | 08 | 03 | 02 |
| 105 | 0.39 | 29 | 08 | 14 | 07 | 02 |
| 106 | 0.47 | 29 | 15 | 14 | 04 | 03 |
| 107 | 0.47 | 29 | 17 | 15 | 01 | 05 |
| 108 | 0.47 | 29 | 01 | 27 | 11 | 03 |
| 109 | 0.47 | 29 | 12 | 35 | 01 | 03 |
| 110 | 0.47 | 29 | 06 | 37 | 08 | 04 |
| 111 | 0.47 | 29 | 12 | 38 | 13 | 06 |
| **112** | **1.89** | **29** | **16** | **44** | **07** | **02** |
| 113 | 0.47 | 29 | 16 | 44 | 11 | 03 |
| 114 | 0.47 | 29 | 06 | 45 | 11 | 03 |
| 115 | 0.47 | 30 | 06 | 18 | 07 | 02 |
| 116 | 0.47 | 30 | 07 | 41 | 13 | 05 |
| 117 | 0.47 | 30 | 04 | 44 | 04 | 06 |
| 118 | 0.47 | 30 | 06 | 44 | 11 | 03 |
| 119 | 0.47 | 30 | 06 | 50 | 13 | 02 |
| 120 | 0.47 | 30 | 05 | 55 | 04 | 02 |
| 121 | 0.47 | 30 | 07 | 58 | 10 | 02 |
| 122 | 0.47 | 31 | 15 | 07 | 04 | 06 |
| 123 | 0.47 | 31 | 16 | 44 | 13 | 03 |
| 124 | 0.47 | 31 | 02 | 50 | 07 | 03 |
| 125 | 0.47 | 31 | 02 | 51 | 13 | 06 |
| 126 | 0.47 | 31 | 07 | 51 | 01 | 05 |
| 127 | 0.47 | 32 | 07 | 07 | 13 | 06 |
| 128 | 0.47 | 32 | 03 | 18 | 03 | 03 |
| 129 | 0.47 | 32 | 01 | 27 | 04 | 03 |
| 130 | 0.47 | 32 | 02 | 27 | 13 | 06 |
| 131 | 0.47 | 32 | 17 | 27 | 13 | 03 |
| 132 | 0.94 | 32 | 04 | 35 | 01 | 03 |
| 133 | 0.47 | 32 | 12 | 39 | 16 | 05 |
| 134 | 0.94 | 32 | 07 | 49 | 07 | 02 |
| 135 | 0.47 | 32 | 07 | 49 | 13 | 06 |
| 136 | 0.47 | 32 | 04 | 53 | 11 | 03 |
| 137 | 0.47 | 32 | 06 | 57 | 07 | 03 |
| 138 | 0.47 | 33 | 07 | 07 | 01 | 05 |
| 139 | 0.47 | 33 | 08 | 14 | 07 | 03 |
| 140 | 0.47 | 33 | 06 | 37 | 10 | 05 |
| 141 | 0.47 | 33 | 12 | 39 | 11 | 03 |

**Table S1**. *(cont.)*

| **Haplotype Nº** | **Relative frequency (%)** | **HLA alleles** | | | | |
| --- | --- | --- | --- | --- | --- | --- |
|  |  | **A*** | **Cw*** | **B*** | **DRB1*** | **DQB1*** |
| 142 | 0.47 | 33 | 17 | 41 | 07 | 02 |
| 143 | 0.47 | 33 | 02 | 44 | 07 | 02 |
| 144 | 0.47 | 66 | 17 | 53 | 04 | 05 |
| 145 | 0.47 | 68 | 07 | 07 | 15 | 06 |
| 146 | 0.94 | 68 | 07 | 15 | 04 | 03 |
| 147 | 0.94 | 68 | 04 | 35 | 13 | 06 |
| 148 | 0.47 | 68 | 12 | 35 | 11 | 05 |
| 149 | 0.47 | 68 | 06 | 37 | 10 | 05 |
| 150 | 0.47 | 68 | 02 | 44 | 09 | 03 |
| 151 | 0.94 | 68 | 07 | 49 | 15 | 06 |
| 152 | 0.47 | 68 | 06 | 50 | 11 | 05 |
| 153 | 0.47 | 68 | 15 | 51 | 04 | 03 |
| 154 | 0.47 | 68 | 04 | 53 | 13 | 06 |
| 155 | 0.47 | 68 | 07 | 58 | 07 | 02 |
| 156 | 0.47 | 68 | 07 | 78 | 16 | 05 |
| 157 | 0.47 | 80 | 12 | 44 | 09 | 03 |
